# Fermented cordyceps powder alleviates silica-induced pulmonary inflammation and fibrosis in rats by regulating the Th immune response

**DOI:** 10.1186/s13020-023-00823-8

**Published:** 2023-10-12

**Authors:** Shuangshuang Pu, Zhifeng Yang, Xiaofeng Zhang, Ming Li, Na Han, Xiaohan Yang, Jin He, Gongchang Yu, Xiangjing Meng, Qiang Jia, Hua Shao

**Affiliations:** 1 Shandong University of Traditional Chinese Medicine, 4655 University Road, Changqing District, Jinan, 250355 Shandong China; 2 Hospital Affiliated to Shandong University of Traditional Chinese Medicine, 16369 Jingshi Road, Lixia District, Jinan, 250014 Shandong China; 3 Shandong Academy of Occupational Health and Occupational Medicine, Shandong First Medical University & Shandong Academy of Medical Sciences, 18877 Jingshi Road, Lixia District, Jinan, 250062 Shandong China; 4 Linyi County Center for Disease Control and Prevention, Linyi County, 91 Yongxing Street, Dezhou, 251500 Shandong China

**Keywords:** Silicosis, Silica, Fermented cordyceps powder, CD4^+^ Th cells

## Abstract

**Background:**

Silicosis is an important occupational disease caused by inhalation of free silica and is characterized by persistent pulmonary inflammation, subsequent fibrosis and lung dysfunction. Until now, there has been no effective treatment for the disease due to the complexity of pathogenesis. Fermented cordyceps powder (FCP) has a similar effect to natural cordyceps in tonifying the lung and kidney. It has started to be used in the adjuvant treatment of silicosis. This work aimed to verify the protective effects of FCP against silicosis, and to explore the related mechanism.

**Methods:**

Wistar rats were randomly divided into four groups including the saline-instilled group, the silica-exposed group, the silica + FCP (300 mg/kg) group and the silica + FCP (600 mg/kg) group. Silicosis rat models were constructed by intratracheal instillation of silica (50 mg). Rats in the FCP intervention groups received the corresponding dose of FCP daily by intragastric gavage. Rats were sacrificed on days 7, 28 and 56 after treatment, then samples were collected for further analysis.

**Results:**

FCP intervention reduced the infiltration of inflammatory cells and the concentration of interleukin-1β (IL-1β), interleukin-6 (IL-6), tumor necrosis factor-α (TNF-α) and transforming growth factor-β1 (TGF-β1) at days 7, 28, 56, and decreased the expression of collagen, α-smooth muscle actin (α-SMA) and fibronectin (FN) at days 28 and 56 in the lung of silicosis rats. FCP also decreased the immune response of Th1 and Th17 at days 7, 28, 56 and inhibited the enhancement of the Th2 response at day 56.

**Conclusions:**

FCP intervention could alleviate silica-induced pulmonary inflammation and fibrosis, the protective effect may be achieved by reducing Th1 and Th17 immune responses and inhibiting the enhancement of the Th2 response.

**Supplementary Information:**

The online version contains supplementary material available at 10.1186/s13020-023-00823-8.

## Introduction

Silicosis is a pulmonary fibrotic occupational disease, endemics of which have been reported worldwide, especially in developing countries [1]. Silicosis is caused by the inhalation of free silica particles. Once inhaled, the silica particles cannot be completely removed. The deposited silica particles in the alveolus can cause persistent pulmonary inflammation, aberrant tissue repair and subsequently irreversible fibrosis, even the workers leaving the exposing environment. The molecular mechanism of silicosis is not fully elucidated. It is widely believed that it is the result of a variety of interacting mechanisms such as macrophage activation, release of cytokines and chemokines, oxidative stress response, cell apoptosis/pyroptosis, activation of adaptive immune system and so on [2, 3].

CD4^+^ T helper cells (Th) are important compositions of the adaptive immune system. They can differentiate into effector T (Th1, Th2, Th17) and regulatory T (Treg) cells according to the specific microenvironment. Th cells are involved in the progress of silicosis due to their clear role in inflammation and fibrosis [4, 5]. Th1 cells that are regulated by the T-box express in T cells (T-bet) can release characteristic inflammatory cytokines interferon-γ (IFN-γ) that promote pulmonary inflammation [6]. Th2 cells, regulated by the GATA-binding protein 3 (GATA3), can secrete interleukin-4 (IL-4) and promote pulmonary fibrosis in the later fibrotic stage [7]. The Th17 and Treg lymphocytes were regulated by the transcription factor of related orphan receptor γt (RORγt) and forkhead box P3 (Foxp3) respectively. Th17 cells secrete interleukin-17A (IL-17A) and are related to both pro-inflammatory and pro-fibrotic effects [8]. Tregs produce transforming growth factor-β (TGF-β) and interleukin-10 (IL-10), and play a crucial part in maintaining immune homeostasis [9].

Increasing evidence has demonstrated the effective effect of Traditional Chinese Medicine (TCM) on silicosis. Cordyceps sinensis is a valued TCM that has medicinal functions to replenish the kidney, soothe the lung, and modulate body immunity [10, 11]. Due to the scarcity and expensive cost of Cordyceps sinensis, the fermented product of Cordyceps sinensis mycelium, i.e. fermented cordyceps powder (FCP), has become a major substitute for the natural species. Commercial products of FCP such as Jinshuibao capsule, have been widely used in clinical treatment of diseases. For example, diabetic nephropathy [12], chronic obstructive pulmonary disease (COPD) [13], idiopathic pulmonary fibrosis (IPF) [14], even Corona Virus Disease 2019 (COVID-19) [15]. Furthermore, FCP has been used as a supplementary drug to treat silicosis [16], but the underlying molecular mechanisms have not been fully elucidated.

To explore the beneficial effects of FCP on silicosis rats and the possible molecular mechanisms, we constructed silicosis rat model by intratracheal instillation of silica and intervened with FCP. We examined the changes of inflammation and fibrosis in the lung, the proportion of the Th1, Th2, Th17 and Treg subsets in peripheral blood (PB) lymphocytes. We examined the expression of T-bet, GATA3, RORγt and Foxp3 mRNA and protein in cells of bronchoalveolar lavage fluid (BALF) and lung sections.

## Materials and methods

### Silicon dioxide

Silicon dioxide (size distribution: 80% between 1 and 5 μm, 99% between 0.5 and 10 μm) was obtained from Sigma Co. (St. Louis, MO, USA). The particles were crushed for 3 h and then suspended in saline at a final concentration of 50 mg/mL. The silicon dioxide suspensions were autoclaved and mixed well before use.

### Compositions determination of Fermented cordyceps powder

Fermented cordyceps powder (FCP) provided by Jiangxi Sino Pharmaceutical Co. LTD. (Nanchang, China), is a fermentation product of Paecilomyces hepialid (Cs-4) purified from Cordyceps sinensis (approval number: National medicine approval Z10890021; Factory batch number: 19090438). FCP is a light brown powder with a special aroma of lactones and a slightly bitter taste.

According to the quality standard of FCP in the 2020 edition of the Chinese Pharmacopoeia, the compositions of adenosine, guanosine, uridine and ergosterol were determined by high performance liquid chromatography (HPLC) (Agilent 1260 Infinity II). The standard curve method was chosen for quantification and the peak area and concentration were proportional. The dilutions of the mixed standard of adenosine, guanosine and uridine were 25, 12.5, 6.25, 0.25 and 0.125 µg/mL. The dilutions of ergosterol standard were 40, 20, 8.0, 4.0, and 0.8 µg/mL. References of adenosine (YZ-110879), guanosine (SG9310), uridine (SU8080) and ergosterol (YZ-111845) were purchased from Solarbio (Beijing, China).

For preparation of test samples for the determination of adenosine, guanosine and uridine, 5 mg of FCP was accurately weighed and 1 mL of 70% methanol was added. After weighing, the mixture was sonicated (power 250 W, frequency 40 kHz) for 20 min, removed to cool, and then reweighed with 70% methanol. The mixture was shaken, filtered. A total of 500 μL was taken to dry and dissolved by adding 500 μL of water. The samples was tested with HPLC and chromatograms were obtained.

For preparation of the test sample for the determination of ergosterol, 2 mg of FCP was accurately weighed and 1 mL of methanol was added. The mixture was weighed, sonicated (power 500 W, frequency 40 kHz) for 60 min, cooled, and reweighed with methanol. The mixture was filtered for testing, and chromatograms were recorded.

Chromatographic condition: Welch Ultimate column (PLUS C18) 250 × 4.6 mm, 5 μm, the temperature was 25 ℃, the flow rate 1 mL/min, and the sample size was 10 μL. The detection wavelength of adenosine, guanosine and uridine was 260 nm. Mobile phase A was 0.02 M potassium dihydrogen phosphate aqueous solution and mobile phase B was methanol. The detection wavelength of ergosterol was 283 nm. Mobile phase A was water and mobile phase B was methanol.

### Rats and treatment

Healthy male Wistar rats (6–8 weeks old) were obtained from Pengyue Experimental Animal Breeding Co., Ltd. (Jinan, China) and raised in a specific pathogen-free (SPF) animal room (temperature 24 ± 2 ℃, humidity 40–70%). Food and water were taken at will. All animal treatment protocols and procedures were approved by the ethical committee of Shandong Academy of Occupational Health and Occupational Medicine (ethical batch number: SDZFY-EC-A-2020-09). Animal experiments were conducted according to Weatherall (2006) Report and NC3Rs Guidelines.

All animals were divided randomly into 4 groups (*n* = 30/group) including the saline-instilled group (saline), the silica-exposed group (silica), silica-exposed rats that intervened with FCP (300 mg/kg) (silica+ FCP 300) and silica-exposed rats that intervened with FCP (600 mg/kg) (silica+ FCP 600). After 3 days of adaptive feeding, the one-time non-exposure intratracheal instillation method was used for animal modeling. Rats in the saline-instilled group were given 1 mL of sterile saline and rats in the other three groups were given 1 mL of silicon dioxide suspension (50 mg/mL). After 24 h, rats in the two intervention groups received the corresponding dose of FCP daily by intragastric gavage, and rats in the saline-instilled group and the silica-exposed group received sterile saline instead of FCP. On day 7, 28 and 56 after treatment, 10 rats in each group were anesthetized by intraperitoneal injection of pentobarbital sodium and sacrificed. Lung tissues and peripheral blood (PB) were collected for further analysis.

### Histological analysis

Lung tissues were fixed with 4% paraformaldehyde, and embedded in paraffin, then cut into 5 μm sections. Sections were dyed with hematoxylin and eosin (HE) to determine the level of inflammation and dyed with Masson staining solution to observe the expression of collagen fiber.

### Analysis of the Th1, Th2, Th17 and Treg cells by Fluorescence-activated cell sorting (FACS)

Lymphocytes in PB were purified with lymphocyte separation medium (TBD science, Tianjin, China). Then the cells surface were stained with CD3-PerCP/Cy5.5 (Biolegend, 201418, USA), CD4-FITC (Invitrogen, 11-0040-82, USA) and CD25-APC (Biolegend, 202114, USA), followed by intracellular staining with IFN-γ-PE (Biolegend, 507806, USA), IL-4-PE (Biolegend, 511906, USA), IL-17A-PE (Invitrogen, 12–7177-81, USA) and Foxp3-PE (Invitrogen, 12-5773-82, USA) using the Transcription Factor Staining Buffer Set (BD Pharmingen, USA) and protocol. The FACS method was performed with the BD FACSCantoTM IIFlow Cytometer (BD Bioscience, USA). The Th1 subsets were recognized as CD3^+^CD4^+^ and IFN-γ^+^ cells, the Th2 subsets were recognized as CD3^+^CD4^+^ and IL-4^+^ cells. The Th17 subsets were recognized as CD3^+^CD4^+^ and IL-17A^+^ cells, while the CD4^+^ and CD25^+^ Foxp3^+^ strategy was used to recognize Treg subsets.

### Quantitative real-time PCR (qRT-PCR) of BALF cells

BALF was obtained by tracheal injection of sterile saline into the lungs and aspirated back, 2 mL each time for six consecutive times. After centrifuged at 1000 *g* for 10 min at 4 ℃, the precipitation of BALF cells was washed twice in PBS. The total messenger RNA (mRNA) of BALF cells was extracted using the SPARKeasy Cell RNA Rapid Extraction Kit (SPARKjade, AC0205, China). With the SPARKscript II RT Plus Kit (SPARKjade, AG0304, China), the mRNA was reversely transcribed into cDNA. Then qRT-PCR was performed with the 2 × SYBR Green qPCR mix (SPARKjade, AH0104, China) and the Roche LightCycler 480 real-time quantitative PCR instrument (Roch, CH). The primers used are shown in Table1. The 2^–ΔΔCT^ method was used for quantitative analysis with β-actin as a reference gene.

**Table1:** Primer sequences of RT-PCR

Gene name	Primer name	Primer sequences	Product size (bp)	Annealing temperature (℃)
β actin	β actin-F	CCCATCTATGAGGGTTACGC	150	60
	β actin-R	TTTAATGTCACGCACGATTTC		
T-bet	T-bet-F	CAACAACAAGGGGGCTTCCA	104	60
	T-bet-R	GCTCACCGTCATTCACCTCC		
GATA-3	GATA-3-F	CTCTTCCCTCCCAGCAGCCTAC	144	60
	GATA-3-R	AGTACCATCTCGCCGCCACAG		
RORγt	RORγt-F	TGGAGAGTGGAAACTGGGAGAGAC	126	60
	RORγt-R	CAGGCTTGGGAGTTGGACATTGG		
Foxp3	Foxp3-F	ACACGCATGTTCGCCTACTTCA	111	60
	Foxp3-R	TGCTCCCTTCTCACTCTCCACT		

### Immunohistochemical analysis

After deparaffinization and rehydration, tissuesections were treated with sodium citrate buffer (0.01 M) in a pressure cooker for 2 min to retrieve antigens. Then the sections were soaked in 3% hydrogen peroxide for 10 min to remove endogenous peroxidase. Sections were incubated with sheep serum at room temperature for 1 h to block nonspecific binding and then incubated at 4 ℃ overnight with the following primary antibodies: Rabbit anti-T-bet antibody (Proteintech, 13,700-1-AP, 1:400, USA); Mouse anti-Gata3 antibody (abcam, ab282110, 1:2000, UK); Rabbit anti-RORγt antibody (Proteintech, 13,205-1-AP, 1: 200, USA); Rabbit anti-Foxp3 antibody (Novus, NB100-39002, 1:400, USA). Following incubation with horseradish peroxidase (HRP) conjugated secondary antibody (DD13: ivision^™^ Poly-HRP sheep anti-mouse/rabbit secondary antibody, TALENT, China) at room temperature for 30 min, the 3,3-diaminobenzidine tetrahydrochloride substrate chromogen solution was used to detect signals and hematoxylin was used to redye the nucleus. The images were captured and then analyzed by Image J.

### Western blot (WB) analysis

Total protein was extracted from lung tissue using RIPA lysis buffer (SPARKjade, EA0002, China). The concentration of the protein samples was detected with the BCA protein assay kit (SPARKjade, EC0001, China). The proteins were then separated by sodium dodecyl sulfate–polyacrylamide gels and transferred to a polyvinylidene fluoride membrane which was blocked with 5% BSA for 1.5 h at room temperature. Then the membrane was incubated overnight at 4 ℃ with the following specific primary antibodies: anti-β actin (proteintech, 20,536-1-AP, 1:2000, USA); anti-α smooth muscle actin (α-SMA) (cst, 14968 s, 1:2000, USA); antifibronectin (FN) (abcam, Ab45688, 1:5000, UK). After washing with TBST, the membrane was incubated with the goat anti-rabbit IgG (H + L) HRP specific secondary antibody (SPARKjade, EF0002, 1:5000, China) for 1 h at room temperature. The Tanon Automatic Image Analysis System (Tanon 5200, China) was used to detect the protein signal and β-actin served as internal reference. The optical density of the protein bands was analyzed using Image J.

### Analysis of cytokines by enzyme-linked immunoassay (ELISA)

Cytokines interleukin-1β (IL-1β), tumor necrosis factor-α (TNF-α), interleukin-6 (IL-6) and transforming growth factor-β1 (TGF-β1) in lung homogenates were tested using commercial ELISA kits (Biokits, ER01-96, ER03-96, ER02-96, ER10-96, China) according to the kit instructions.

### Statistical analysis

All data were presented as mean ± standard error of the mean (SEM). SPSS (20.0) software was used for statistical analysis. One-way analysis of variance (ANOVA) and the Student–Newman–Keuls (SNK) test were used to analyze differences between groups. *P*-value less than 0.05 was considered difference statistically.

## Results

### Determination of compositions in the FCP sample

**Fig. 1:**
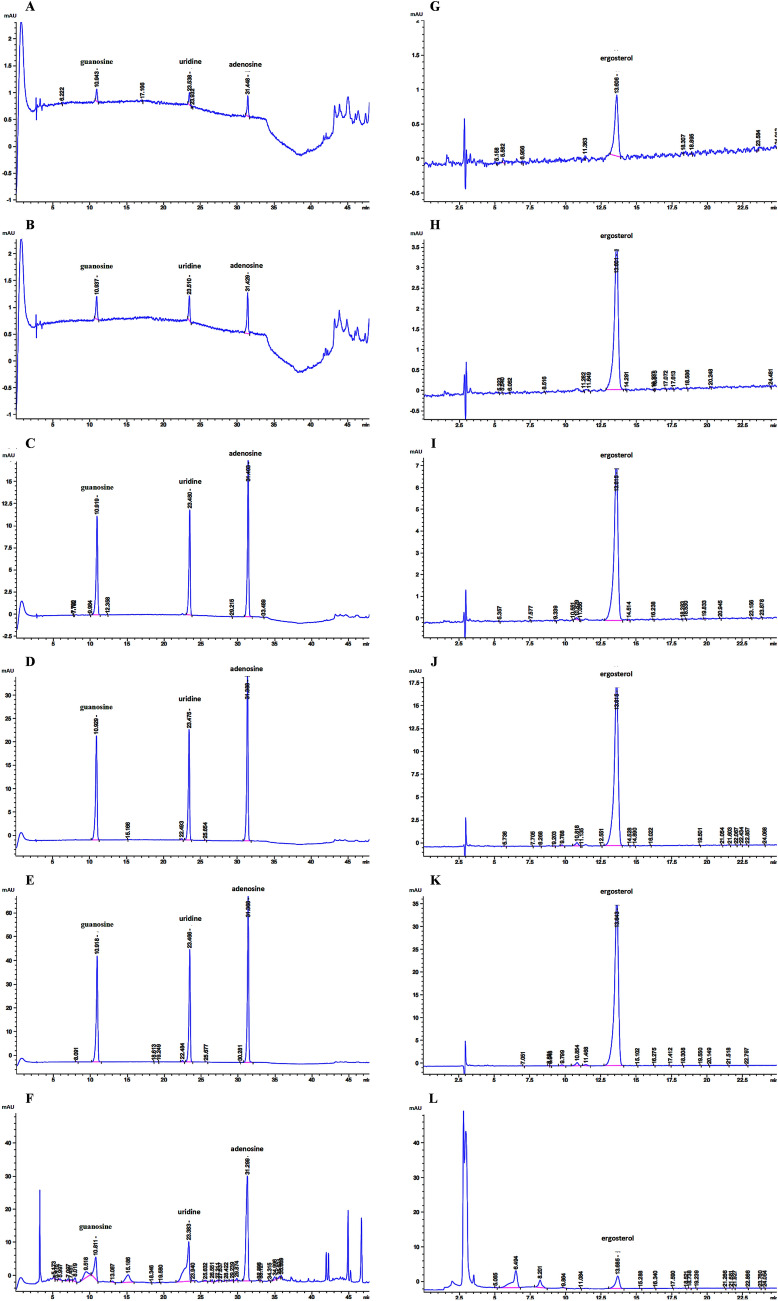

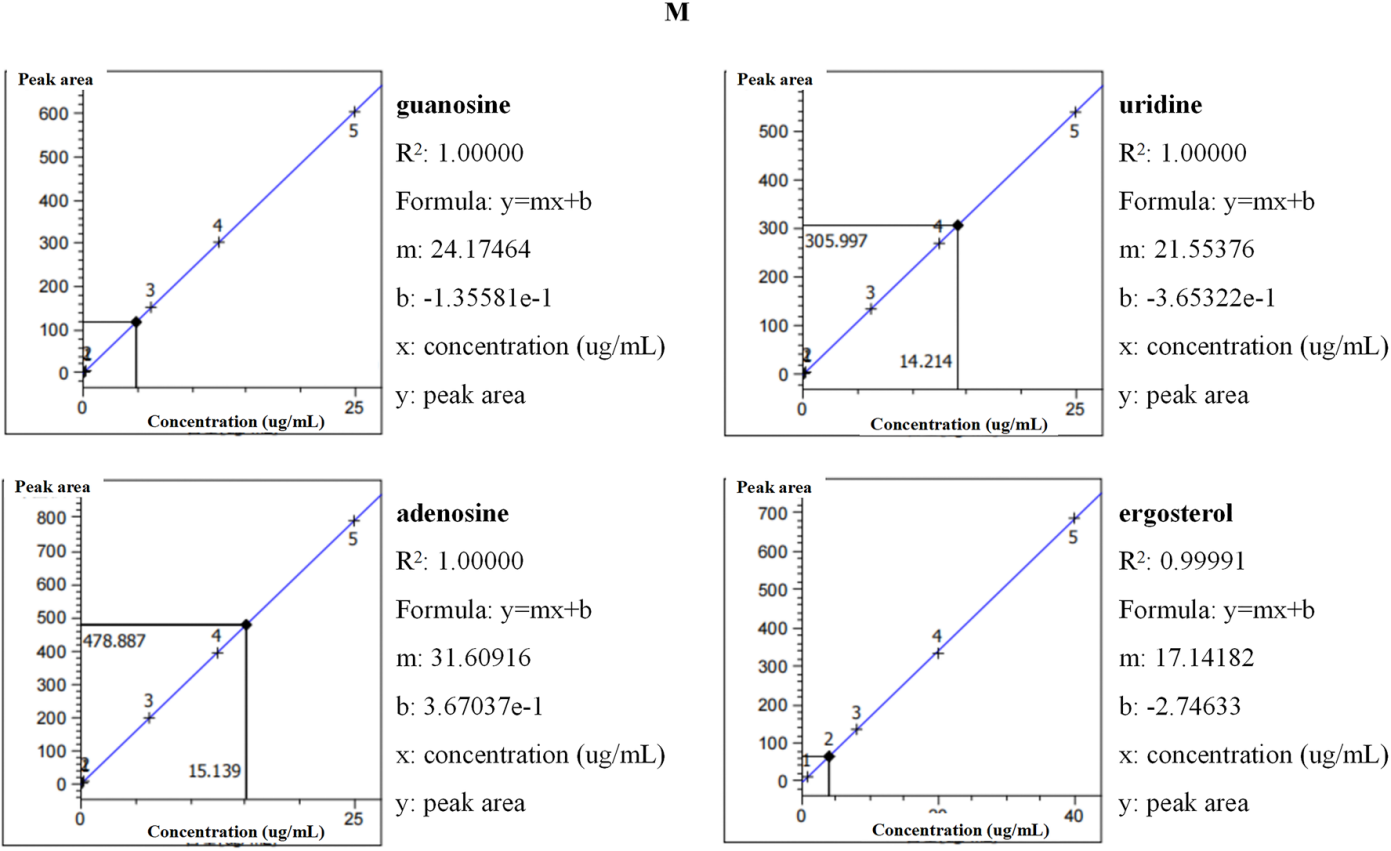
Chromatograms and standard curves. **A**–**E** Chromatograms of various concentrations of mixed standard of guanosine, uridine and adenosine. **F** Detection chromatogram of guanosine, uridine and adenosine in the FCP sample. **G**–**K** Chromatograms of various concentrations of ergosterol standard. **L** Detection chromatogram of ergosterol in the FCP sample. **M** Standard curves for guanosine, uridine, adenosine, and ergosterol

According to the quality standard of FCP in the 2020 edition of Chinese Pharmacopoeia, per 0.33 g FCP, the total amount of guanosine, uridine and adenosine should not be less than 1.6 mg and ergosterol should not be less than 0.66 mg. We determined the peak area of various concentrations of the mixed standard (Fig. 1A–E), the ergosterol standard (Fig. 1G–K), and the FCP sample (Fig. 1F, L) by HPLC. The R^2^ of the four standard curves were all greater than 0.9999 (Fig. 1M). The mass concentration data of FCP were analyzed and the results are shown in Table 2, which fully meets the quality requirements.

**Table 2:** Data analysis of FCP composition

Composition name	Sample peak area	Sample concentration	Sample weight	Sample volume	Sample mass concentration	Quality requirements
	(mAU*s)	(ug/mL)	(mg)	(mL)	(mg/g)	(mg/g)
Guanosine	117.87	4.88	5.00	1.00	0.98	Total amount ≥ 4.8
Uridine	306.00	14.21	5.00	1.00	2.84	
Adenosine	478.89	15.14	5.00	1.00	3.03	
Ergosterol	66.22	4.02	2.00	1.00	2.01	≥ 1.98

### FCP alleviated silica-induced pulmonary inflammation and organic coefficient of the lung

**Fig. 2:**
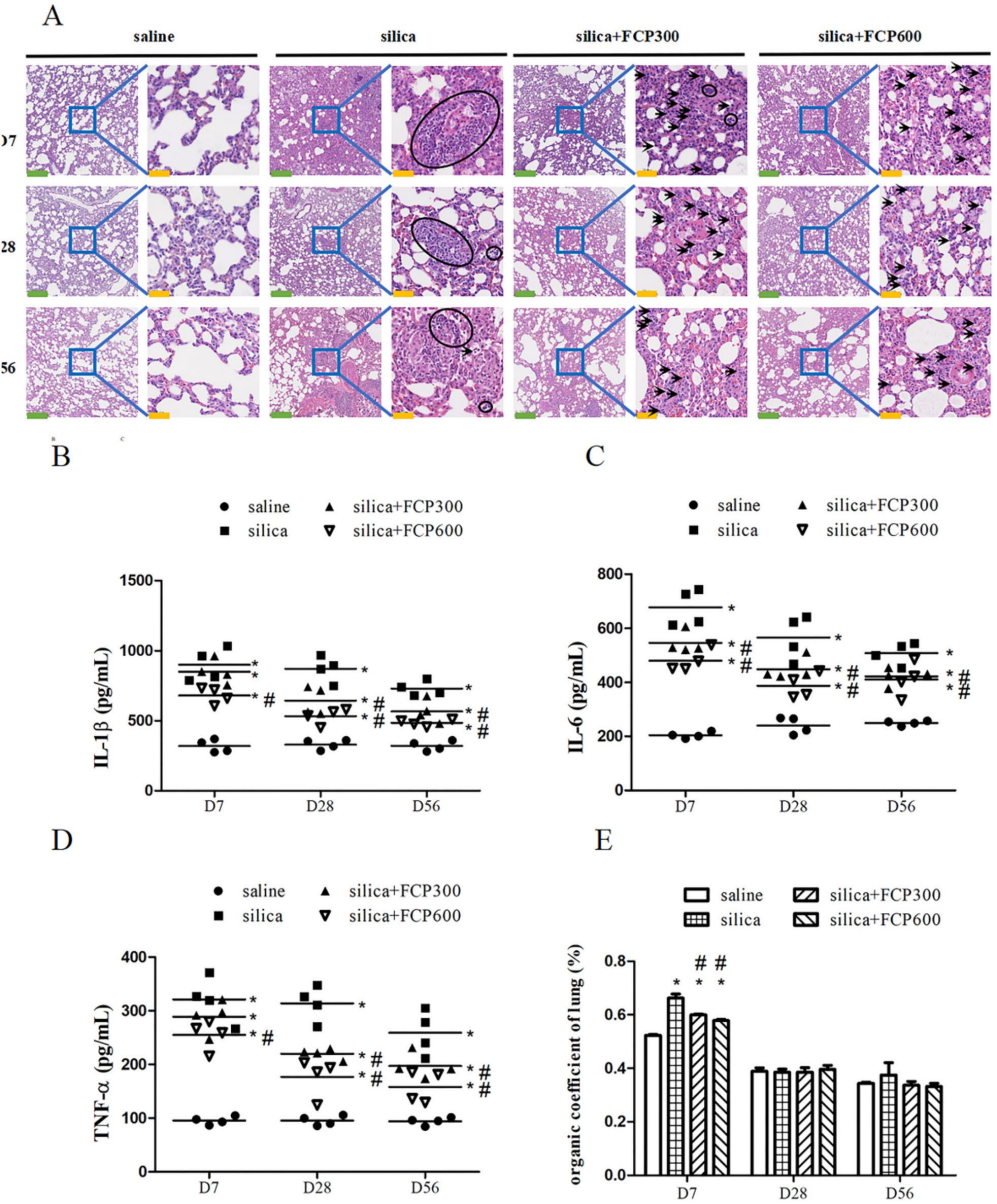
FCP alleviated silica-induced pulmonary inflammation and organic coefficient of lung. **A** HE staining of lung sections at days 7, 28 and 56. The scale for the left figures is 40 × , and the green scale bars in the figures represent 250 μm. The scale for the right figures is 200 × , and the yellow scale bars in the figures represent 50 μm. Black circles represent aggregated inflammatory cells, and black arrows represent scattered inflammatory cells. **B**–**D** ELISA analysis of cytokines IL-1β, IL-6, and TNF-α in lung homogenate. **E** Analysis of the organic coefficient of the lung. Data are presented as mean ± SEM. ^**∗**^*P* < 0.05 compared to saline-instilled group; ^#^ *P* < 0.05 compared to the silica-exposed group. Saline: saline-instilled group; silica: silica-exposed group; silica + FCP 300: silica-exposed rats that were intervened with FCP (300 mg/kg); silica + FCP 600: silica-exposed rats that were intervened with FCP (600 mg/kg)

Results of pulmonary inflammation in rats were shown in Fig. 2A. The lungs of silica-exposed rats had remarkable infiltration of inflammatory cells on days 7, 28 and 56 respectively compared with that of saline-instilled rats. After FCP intervention, these inflammatory cells in the lungs of silica + FCP (300, 600 mg/kg) intervened rats were obviously reduced compared with silica-exposed rats at days 7, 28 and 56.

The concentration of cytokines IL-1β, TNF-α and IL-6 in the lung homogenate were measured by ELISA and the results showed that, the levels of all the three cytokines in the lungs of silica-exposed rats were significantly higher than that in saline-instilled rats at days 7, 28 and 56 (*P* < 0.05). However, compared with that in silica-exposed rats, IL-6 expression in the lungs of silica+ FCP (300, 600 mg/kg) intervened rats decreased significantly at days 7, 28 and 56 (*P* < 0.05). In addition, IL-1β and TNF-α in the lungs of silica+ FCP (300, 600 mg/kg)-intervened rats significantly decreased at days 28 and 56 (*P* < 0.05) as shown in Fig. 2B–D.

Analysis of the organic coefficient of the lung revealed that the index in the silica-exposed group was significantly higher than in the saline-instilled group on day 7 (*P* < 0.05). After FCP intervention, the index in the silica+ FCP (300, 600 mg/kg) groups was significantly reduced compared with the silica-exposed group on day 7 (*P* < 0.05). But at days 28 and 56, no significant difference was found in the index among the experimental groups because of the rapid weight gain of rats, as shown in Fig. 2E.

### FCP alleviated silica-induced pulmonary fibrosis

**Fig. 3:**
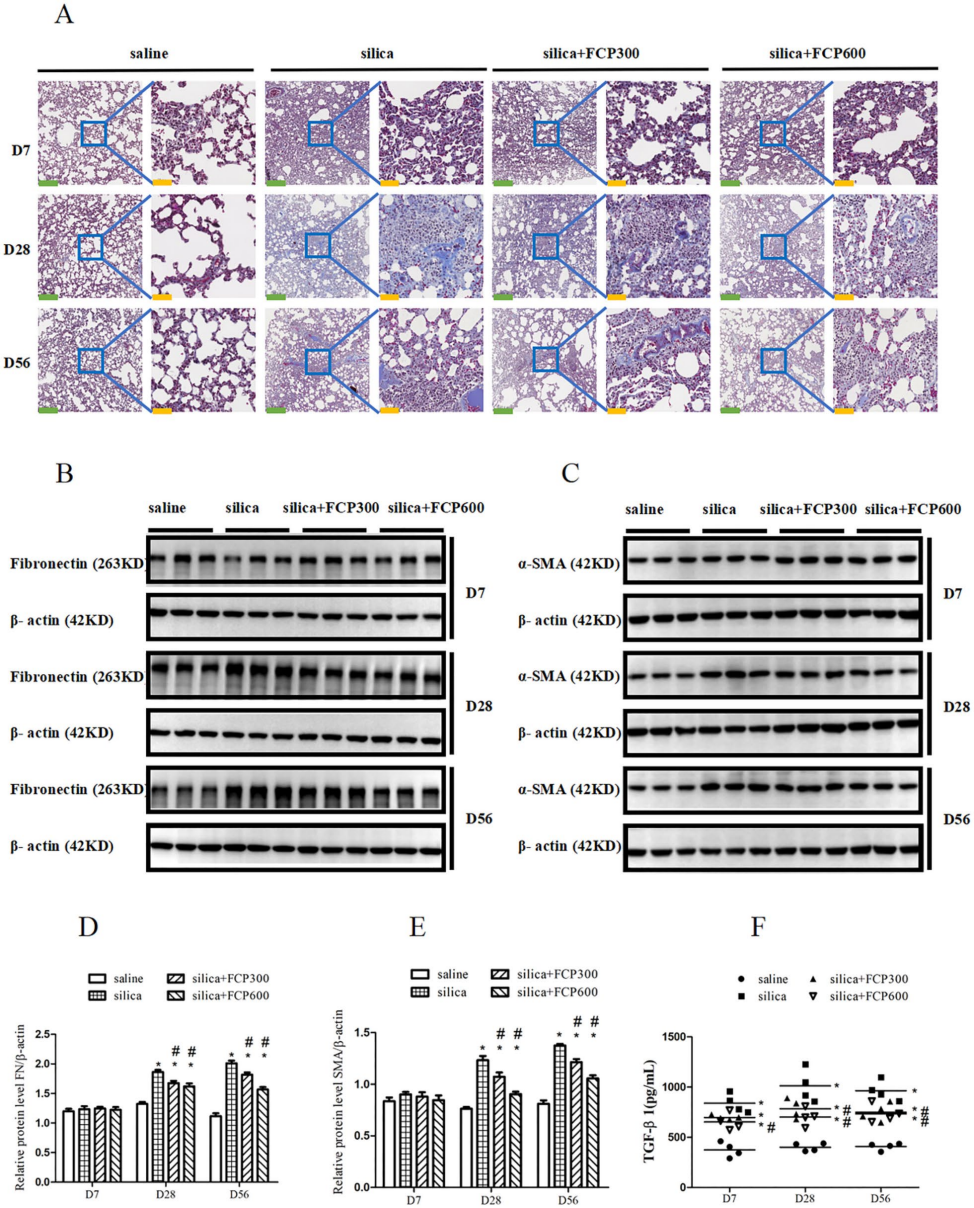
FCP ameliorated silica-induced pulmonary fibrosis. **A** Masson staining of rat lung sections on days 7, 28 and 56. The scale for the left figures is 40 × , and the green scale bars in the figures represent 250 μm. The scale for the right figures is 200 × , and the yellow scale bars in the figures represent 50 μm. **B**, **C** Western blot analysis of FN and α-SMA in lung tissues. **D**, **E** Quantification of the WB bands of FN and SMA, data showed the ratio of the target protein to β-actin. F ELISA analysis of TGF-β1 in lung homogenate. Data are presented as mean ± SEM. ^**∗**^*P* < 0.05 compared to saline-instilled group; ^#^ *P* < 0.05 compared to the silica-exposed group. saline: saline-instilled group; silica: silica-exposed group; silica + FCP 300: silica-exposed rats that were intervened with FCP (300 mg/kg); silica + FCP 600: silica-exposed rats that were intervened with FCP (600 mg/kg)

To explore whether FCP could alleviate silica-induced pulmonary fibrosis, we stained lung sections of rats with Masson. As shown in Fig. 3A, the lungs of silica-exposed rats had remarkable collagen fiber deposition compared to saline-instilled rats on days 28 and 56. After FCP intervention, collagen deposition in the lungs of silica+ FCP (300, 600 mg/kg) intervened rats was obviously reduced compared to silica-exposed rats at days 28 and 56.

We also detected the expression of FN and α-SMA in the lungs by WB. As shown in Fig. 3B–E, compared with saline-instilled rats, the expression of FN and α-SMA significantly increased in the lungs of silica-exposed rats on days 28 and 56 (*P* < 0.05), which was consistent with collagen deposition. However, compared with silica-exposed rats, FN and α-SMA significantly decreased in the lungs of silica+ FCP (300, 600 mg/kg)-intervened rats on days 28 and 56 (*P* < 0.05).

TGF-β1 is a recognized profibrotic cytokine, we detected the levels of TGF-β1 in the lung homogenate by ELISA. As shown in Fig. 3F, the levels of TGF-β1 in the lungs of silica-exposed rats were significantly higher than those in saline-instilled rats at days 7, 28 and 56 (*P* < 0.05). After FCP intervention, TGF-β1 in the lungs of silica+ FCP (300, 600 mg/kg) intervened rats decreased significantly compared to silica-exposed rats on days 28 and 56 (*P* < 0.05).

### FCP reduced the Th1 immune response and inhibited the enhancement of the Th2 immune response in silica-exposed rats.

Th1 and Th2 immune responses play a vital role in the progression of pulmonary inflammation and fibrosis. Therefore, we explored the effects of FCP on the Th1 and Th2 immune response in silica-exposed rats.

**Fig. 4:**
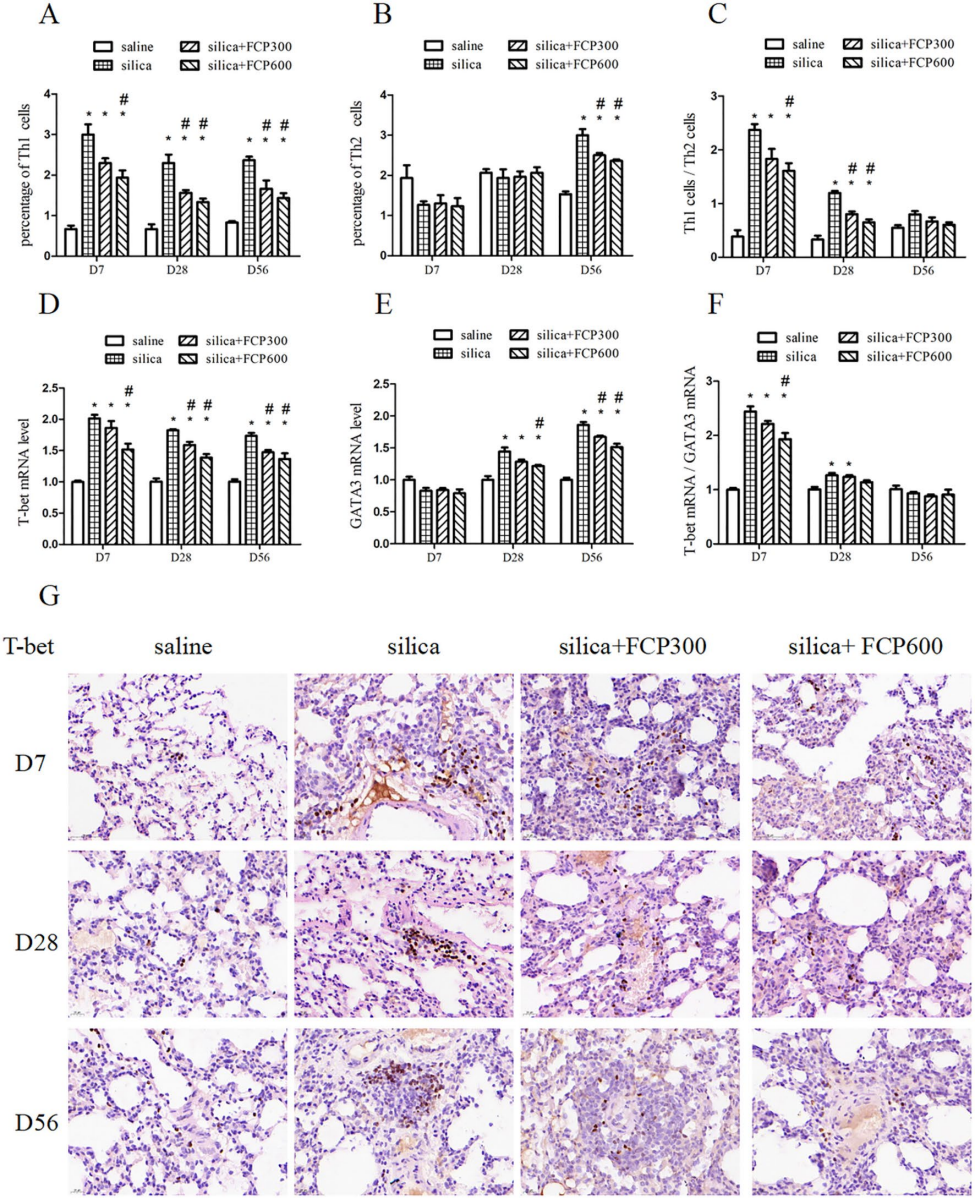

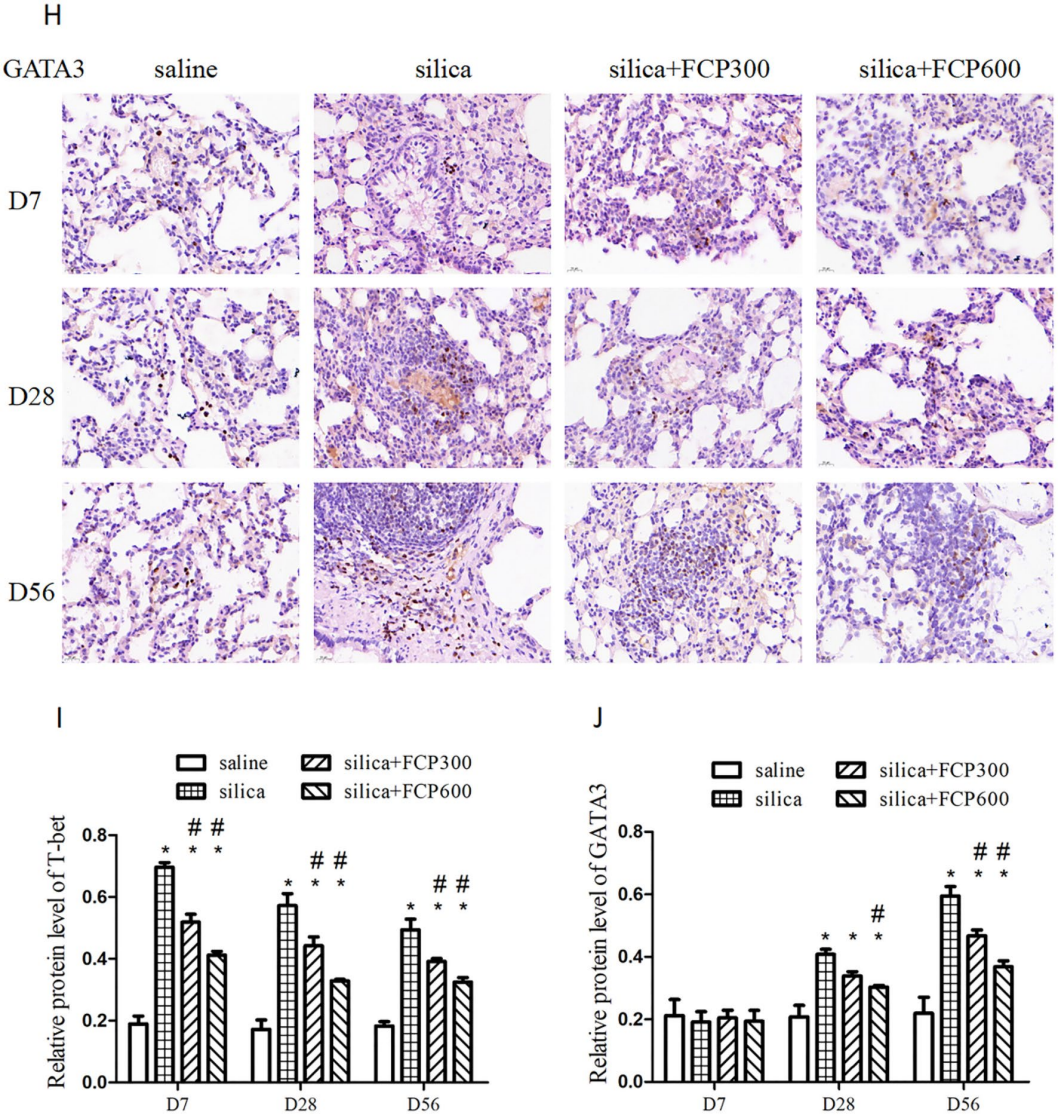
FCP reduced the Th1 immune response and inhibited the enhancement of the Th2 immune response in silica-exposed rats. **A**, **B** The detection of Th1 (CD3^+^CD4^+^IFN-γ^+^) cells and Th2 (CD3^+^CD4^+^IL-4^+^) cells in peripheral blood lymphocytes by flow cytometry. **C** The ratio of Th1 cells to Th2 cells. **D**, **E** mRNA levels of T-bet and GATA3 in BALF cells assayed by qRT-PCR. **F** The ratio of T-bet mRNA to GATA3 mRNA. **G**, **I** Relative protein level of T-bet in lung sections detected by immunohistochemistry. H, **J** The relative protein level of GATA3 in lung sections detected by immunohistochemistry. Data are presented as mean ± SEM. ^**∗**^*P* < 0.05 compared to saline-instilled group; ^#^ *P* < 0.05 compared to the silica-exposed group. saline: saline-instilled group; silica: silica-exposed group; silica + FCP 300: silica-exposed rats that were intervened with FCP (300 mg/kg); silica + FCP 600: silica-exposed rats that were intervened with FCP (600 mg/kg)

IFN-γ is the typical effecter cytokine of Th1 cells. We detected Th1 (CD3^+^CD4^+^IFN-γ^+^) cells in PB lymphocytes by flow cytometry. Furthermore, T-bet is the nuclear transcription factor of Th1 cells. We detected T-bet mRNA in BALF cells and T-bet protein in lung tissue with PCR and immunohistochemical method. As shown in Fig. 4A and D, the proportion of Th1 cells in PB lymphocytes and the expression of T-bet mRNA in BALF cells were significantly higher in the silica-exposed group than that in the saline-instilled group on days 7, 28 and 56 (*P* < 0.05). After the FCP intervention, the percentage of Th1 cells and the expression of T-bet mRNA in the silica + FCP (300, 600 mg/kg) groups were significantly reduced compared to the silica-exposed group at days 28 and 56 (*P* < 0.05). The immunohistochemical results showed that, compared to the saline-instilled group, the expression of the T-bet protein in the silica-exposed group increased significantly on days 7, 28 and 56 (*P* < 0.05). However, compared with the silica-exposed group, the expression of the T-bet protein decreased significantly in the silica + FCP (300, 600 mg/kg) groups on days 7, 28 and 56 (*P* < 0.05), as shown in Fig. 4I.

IL-4 is the typical Th2 cell cytokine. We found that the percentage of Th2 (CD3^+^CD4^+^IL-4^+^) cells in PB lymphocytes was significantly higher in the silica-exposed group than in the saline-instilled group on day 56 (*P* < 0.05). However, the proportion of Th2 cells in the silica + FCP (300, 600 mg/kg) groups decreased significantly on day 56 (*P* < 0.05) compared with that of rats in silica-exposed group (Fig. 4B). GATA3 is the nuclear transcription factor of Th2 cells. As shown in Fig. 4E and J, the expression of GATA3 mRNA in BALF cells and GATA3 protein in lung tissue obviously increased in the silica-exposed group compared to the saline-instilled group on days 28 and 56 (*P* < 0.05), but the expression of GATA3 mRNA and GATA3 protein decreased significantly in the silica+ FCP (300, 600 mg/kg) groups compared to the silica-exposed group on day 56 (*P* < 0.05).

For each rat, we calculated the ratio of Th1 cells to Th2 cells in PB lymphocytes and the ratio of T-bet mRNA to GATA3 mRNA in BALF cells. The ratio of Th1/Th2 cells and T-bet mRNA/GATA3 mRNA was significantly increased in the silica-exposed group compared with the saline-instilled group on days 7 and 28 (*P* < 0.05), but no significant differences were found on day 56. After FCP intervention, the two ratios were significantly inhibited in the silica+ FCP (600 mg/kg) group compared with those in the silica-exposed group on day 7. The Th1/Th2 cells decreased significantly in the silica + FCP groups (300, 600 mg/kg) on day 28 (*P* < 0.05), as shown in Fig. 4C and F.

### FCP attenuated the Th17 immune response but had no significant effect on Treg in silica-exposed rats

**Fig. 5:**
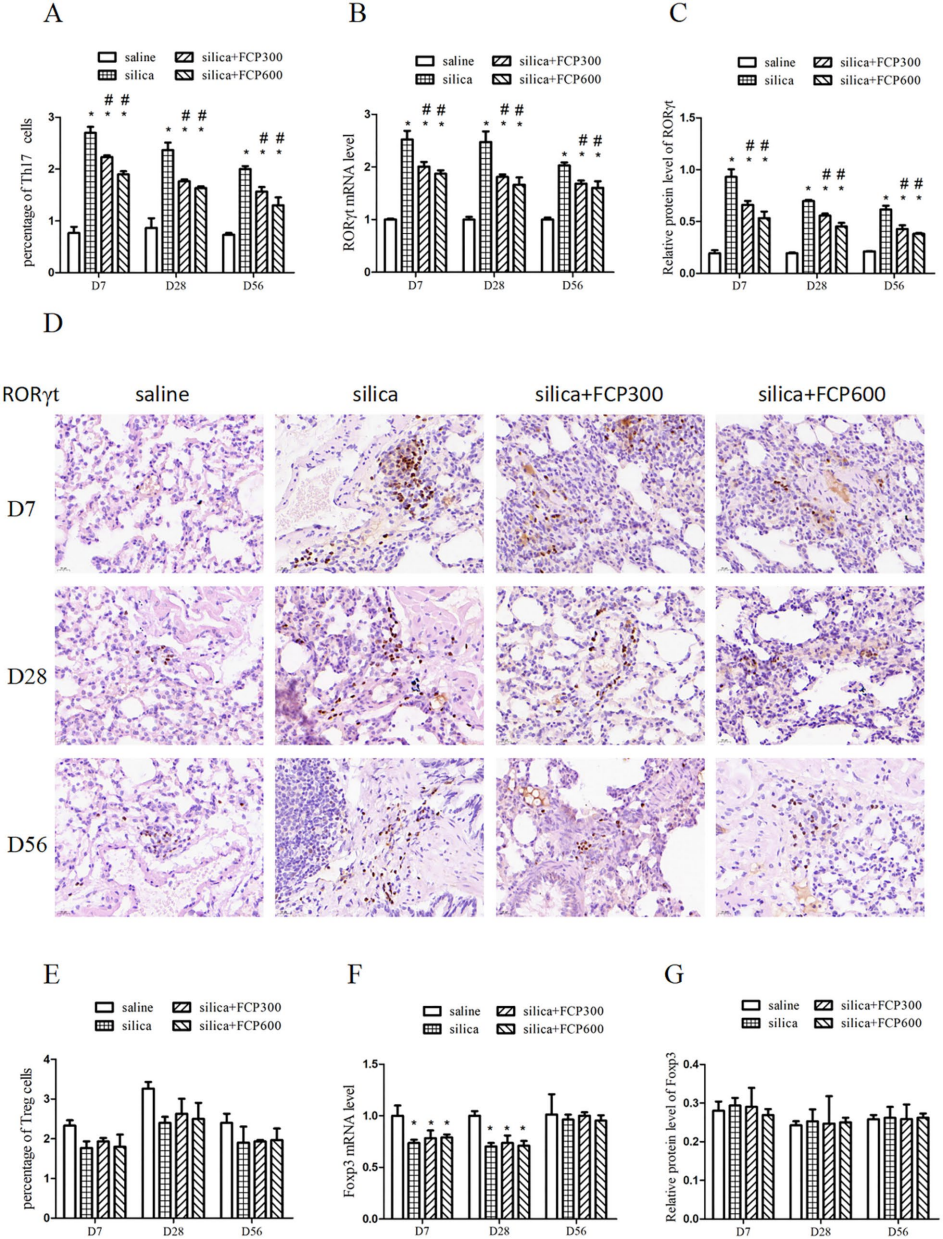
FCP attenuated the Th17 immune response but had no significant effect on Treg in silica-exposed rats. **A** The detection of Th17 cells (CD3^+^CD4^+^IL-17A^+^) in PB lymphocytes by flow cytometry. **B** The mRNA levels of RORγt in BALF cells assayed by qRT-PCR. **C**, **D** The relative level of RORγt in lung sections detected by immunohistochemistry. **E** The detection of Treg cells (CD4^+^CD25^+^Foxp3^+^) in PB lymphocytes by flow cytometry. **F** The mRNA levels of Foxp3 in BALF cells assayed by qRT-PCR. **G** The relative level of Foxp3 in lung sections detected by immunohistochemistry. Data are presented as mean ± SEM. ^**∗**^*P* < 0.05 compared to saline-instilled group; ^#^ *P* < 0.05 compared to the silica-exposed group. saline: saline-instilled group; silica: silica-exposed group; silica + FCP 300: silica-exposed rats that were intervened with FCP (300 mg/kg); silica + FCP 600: silica-exposed rats that were intervened with FCP (600 mg/kg)

The Th17 and Treg immune responses are involved in the progression of pulmonary inflammation and fibrosis. We examined Th17 cells (CD4^+^CD25^+^IL-17A^+^) in PB lymphocytes by flow cytometry, RORγt mRNA in BALF cells by PCR, and RORγt protein in lung tissue by immunohistochemistry. The results displayed that the proportion of Th17 cells (CD4^+^CD25^+^IL-17A^+^), the expression level of RORγt mRNA and RORγt protein increased significantly in the silica-exposed group at days 7, 28 and 56 (*P* < 0.05). These effects decreased significantly in the silica + FCP (300, 600 mg/kg) groups compared with the silica-exposed group on days 7, 28 and 56 (*P* < 0.05), as shown in Fig. 5A–C.

Foxp3 is the specific nuclear transcription factor for Treg cells. We detected Treg cells (CD4^+^CD25^+^Foxp3^+^) in PB lymphocytes, Foxp3 mRNA in BALF cells, and Foxp3 protein expression in lung tissue. The results showed that the expression of Foxp3 mRNA in BALF cells was significantly decreased in the silica-exposed group compared to saline-instilled group on days 7 and 28 (*P* < 0.05) (Fig. 5F), the percentage of Treg cells in the silica-exposed group also showed a decreasing trend (Fig. 5E), but no significant improvement was found after FCP intervention. There was no significant difference in Foxp3 protein expression among all groups by immunohistochemical method (Fig. 5G).

## Discussion

To study the pathogenesis of pulmonary inflammation and fibrosis caused by silica and explore potential therapeutic medicines for the treatment of this disease, we first established an experimental rat silicosis model using 1 mL of 50 mg/mL silica suspension with non-exposure intratracheal instillation method. The method is the most commonly used protocol for silicosis due to its simple operation, easy control of dust dose and low mortality rate. For the basis for selecting 7, 28, and 56 days, we referred to a study [17] which suggested a protocol (dose and time of silica exposure in tracheal instillation) to establish silicosis models in different stages. The study showed that silica exposure at 50 mg for 5 or 10 days in rats is an appropriate condition of inflammatory stage. Exposure to 50 mg of silica for at least 30 days in rats is suitable for fibrotic formation. Therefore, we selected a time point of 7 days to simulate the inflammatory phase and selected 28 and 56 days to simulate the fibrotic silicosis process. Meanwhile, we intervened the silica-exposed rats with FCP, a fermentation product of Cordyceps sinensis mycelium that has multiple beneficial bioactivities [10, 11], to investigate its protective effects and possible mechanisms in silicosis rats.

Once silica particles enter the lungs, alveolar macrophages (AMs), the first defense of innate immunity, phagocytize silica particles and become activated. Activated AMs could produce pro-inflammatory cytokines which play an important role in the recruitment of inflammatory cells, including macrophages, neutrophils, and lymphocytes to the lung. Extra inflammatory cytokines and cells ultimately lead to uncontrolled cycles of pulmonary inflammation and tissue damage [18, 19]. Persistent pulmonary inflammation leads to aberrant tissue repair and subsequently irreversible fibrosis. TGF-β1, the most potent profibrotic cytokine, could induce fibroblasts and epithelial cells to activate, proliferate and eventually transdifferentiate into myofibroblasts, which could generate compositions of the extracellular matrix, such as collagen fiber, FN and α-SMA [20, 21]. In our study, we found high levels of IL-1β, IL-6, TNF-α and remarkable inflammatory cell infiltration in the lung of silica-exposed rats compared with control rats on days 7, 28 and 56. These result was consistent with a cluster analysis in rat and mouse silicosis models which demonstrated that lung inflammation worsened significantly during the inflammatory phase and continued until the later fibrotic phase [17]. The silica particles cannot be digested by macrophages, and they were continuously released and then phagocytized, which lead to the persistent inflammation in the pathophysiological process of silicosis. Our results also presented that TGF-β1 was higher in the lung homogenate of silica-exposed rats than the controls on days 7, 28 and 56. From day 28 the expression of FN, α-SMA and collagen fiber increased in the lung of silica-exposed rats. The elevation of inflammatory and fibrotic markers indicated that our rat silicosis model was established successfully.

In the study, we intervened in silicosis rats with two doses of FCP, 300 mg/kg was the normal dose, which was equal to the human dose based on the body surface area calculation method, and 600 mg/kg as a high dose. The intervention results indicated that FCP alleviated the silica-induced infiltration of inflammatory cells and reduced the high expression of IL-1β, TNF-α, IL-6 on days 7, 28 and 56 in a dose-dependent manner. The FCP intervention also decreased the expression of TGF-β1 and fibrotic proteins obviously. These results in silicosis rats were consistent with the reducing effect of FCP on TNF-α and TGF-β1 in the serum of silicosis patients [16], and confirmed that FCP had a marked inhibitory effect on silica-induced inflammation and fibrosis.

During the progression of pulmonary inflammation and fibrosis caused by silica, the CD4^+^ cell subsets played a vital role [4, 22]. Th1 cells that are regulated by T-bet, usually function as proinflammatory cells by secreting the hallmark IFN-γ [6]. Th2 cells that are regulated by GATA3, secrete the characteristic cytokine IL-4 and play a crucial part in the later fibrotic stage [7]. Previous studies have shown that Th1/Th2 imbalances participate in silicosis, and Th cells shift from Th1 predominance in the inflammatory stage to Th2 predominance during the fibrotic stage [23, 24]. In this study, we found that the proportion of Th1 (CD3^+^CD4^+^IFN-γ^+^) cells in PB lymphocytes, the expression of T-bet mRNA in BALF cells and the T-bet protein in the lung were significantly increased in silica-exposed rats in the early inflammatory phase(7 day) and later in fibrotic progression (28 and 56 day). We also discovered that the proportion of Th2 cells (CD3^+^CD4^+^IL-4^+^) increased significantly in silica-exposed rats on day 56, while the expression of GATA3 mRNA and protein increased significantly on days 28 and 56. The results showed that Th2 cell immunity was gradually enhanced with the progression of fibrosis leading to a decrease of Th1/Th2. After intervention, FCP obviously inhibited Th1 cell immunity throughout the development of silicosis and inhibited the enhancement of Th2 cell immunity in later fibrotic progression in a dose-dependent manner, while effectively correcting the silica-induced Th1/Th2 imbalance.

Th17 cells are characterized by the ability to produce IL-17A, which can recruit inflammatory cells and stimulate them to secrete a large number of inflammatory cytokines [25]. Wilson et al. found that, compared with WT mice, IL-17A deficiency significantly reduced bleomycin-induced collagen expression in the lung and BALF of il17a^−/−^ mice [26]. Furthermore, Chen et al. demonstrated that IL-17A neutralization could delay both the progression of silicosis inflammation and fibrosis in C57BL/6 mice [8]. Therefore, Th17 cells were determined to be associated with proinflammatory and profibrotic effects. Our results presented that the proportion of Th17 (CD3^+^CD4^+^IL-17A^+^) cells and the expression of RORγt mRNA and protein were significantly enhanced in silica-exposed rats at days 7, 28 and 56, and FCP intervention could effectively inhibit Th17 polarization in a dose-dependent manner.

Previous research has confirmed that Treg defects enhance the infiltration of inflammatory cells in the early stage of silicosis, but delay the fibrosis process in the late stage [27]. In addition, Treg also can regulate the homeostasis of effector T cells, and Treg cell injection has been shown to reduce collagen-induced joint inflammation by reducing the Th1/Th2 ratio [28]. In this study, we found the expression of Foxp3 mRNA in BALF cells was decreased significantly in the silica-exposed rats on days 7 and 28, but FCP intervention did not improve this marker. The effect of FCP on the reduction of Th1/Th2 ratio may not be realized through the increase of Treg.

The main chemical composition of FCP are similar to that of Cordyceps sinensis, such as polysaccharide, nucleoside, sterol and so on [29]. It is often used as a substitute for natural cordyceps sinensis. Currently, the content of adenosine, guanosine, uridine and ergosterol is specified as the quality criteria for FCP included in the 2020 edition of Chinese Pharmacopoeia [30]. In this study, we determined compositions of adenosine, guanosine, uridine and ergosterol by HPLC, the results showed that the quality criteria were met. Previous studies of Cordyceps sinensis on fibrosis and immune function may provide evidence for the effects of the FCP intervention presented in this study. Cordyceps sinensis has been found to decrease α-SMA expression in the renal fibrosis mice model and hydroxyproline expression in the pulmonary fibrosis mice model [31, 32]. After treatment with cordyceps sinensis extract, the serum levels of IL-4 and IL-17, which are characteristic cytokines of Th2 and Th17 immunity, were significantly decreased in cobalt-60 exposed mice [33]. Das et al. concluded that Cordyceps sinensis-derived constituents could regulate the induction of Th1 and Th2 through the signal pathway activated by C-type lectin receptors (CLRs) and Toll-like receptors (TLRs) [34]. Furthermore, increasing evidence showed that Cordyceps sinensis and its compounds have bidirectional regulatory capabilities, which can both enhance the immune deficiency and control the overimmunity [34, 35]. Our results indicate that, in the overactive immunity induced by silica particles, FCP significantly decreased Th1 and Th17 responses and inhibited the enhancement of Th2 response, which might explain its protective anti-inflammatory and antifibrotic effects. This study confirmed the intervention effect of FCP on silicosis, however the effect of specific components need to be further studied.

## Conclusion

In this study, we successfully established the silicosis rat model and dynamically observed the early pulmonary inflammation and late fibrosis process on day 7, 28 and 56. After the FCP intervention, silica-induced inflammation and fibrosis were significantly ameliorated, which may be achieved by reducing Th1 and Th17 immune responses and inhibiting the enhancement of the Th2 response. We confirmed the protective effect and possible mechanism of FCP in the development of silicosis, which could provide important basis for early intervention and treatment of silicosis.

### Supplementary Information

**Additional file 1. **Flow cytometry of CD4^+^ T cell subsets in peripheral blood lymphocytes of rats. **Figure S1.** Th1 (CD3^+^CD4^+^IFN-γ^+^) cells. **Figure S2.** Th2 (CD3^+^CD4^+^IL-4^+^) cells. **Figure S3**. Th17 (CD3^+^CD4^+^IL-17A^+^) cells. **Figure S4.** Treg (CD4^+^CD25^+^Foxp3^+^) cells.

## Data Availability

All data generated or analysed during this study are included in this published article and in Additional file 1.

## Abbreviations

FCP: Fermented cordyceps powder
Th: T helper cells
Treg: Regulatory T
T-bet: T-box express in T cells
GATA3: GATA-binding protein 3
RORγt: Related orphan receptor γt
Foxp3: Forkhead box P3
COPD: Chronic obstructive pulmonary disease
IPF: Idiopathic pulmonary fibrosis
COVID-19: Corona Virus Disease 2019
PB: Peripheral blood
BALF: Bronchoalveolar lavage fluid
HPLC: High performance liquid chromatography
SPF: Specific pathogen free
HE: Hematoxylin and eosin
FACS: Fluorescence-activated cell sorting
HRP: Horseradish peroxidase
ELISA: Enzyme-linked immunoassay
IL-1β: Interleukin-1β
IL-6: Interleukin-6
TNF-α: Tumor necrosis factor-α
TGF-β1: Transforming growth factor-β1
IFN-γ: Interferon-γ
IL-4: Interleukin-4
IL-17A: Interleukin-17A
IL-10: Interleukin-10
α SMA: α Smooth muscle actin
FN: Fibronectin
SEM: Standard error of the mean
ANOVA: One-way analysis of variance
SNK: Student–Newman–Keuls
WB: Western Blot
AMs: Alveolar macrophages
CLRs: C-type lectin receptors
TLRs: Toll-like receptors
