# Supplementary material for: Fermented cordyceps powder alleviates silica-induced pulmonary inflammation and fibrosis in rats by regulating the Th immune response
Source: Chin Med. 2023 Oct 12;18:131. doi: 10.1186/s13020-023-00823-8 (PMC10571334; doi:10.1186/s13020-023-00823-8)
Supplement: Supplementary file 1 — Additional file 1. Flow cytometry of CD4+ T cell subsets in peripheral blood lymphocytes of rats. Figure S1. Th1 (CD3+CD4+IFN-γ+) cells. Figure S2. Th2 (CD3+CD4+IL-4+) cells. Figure S3. Th17 (CD3+CD4+IL-17A+) cells. Figure S4. Treg (CD4+CD25+Foxp3+) cells. [file 13020_2023_823_MOESM1_ESM.pdf]

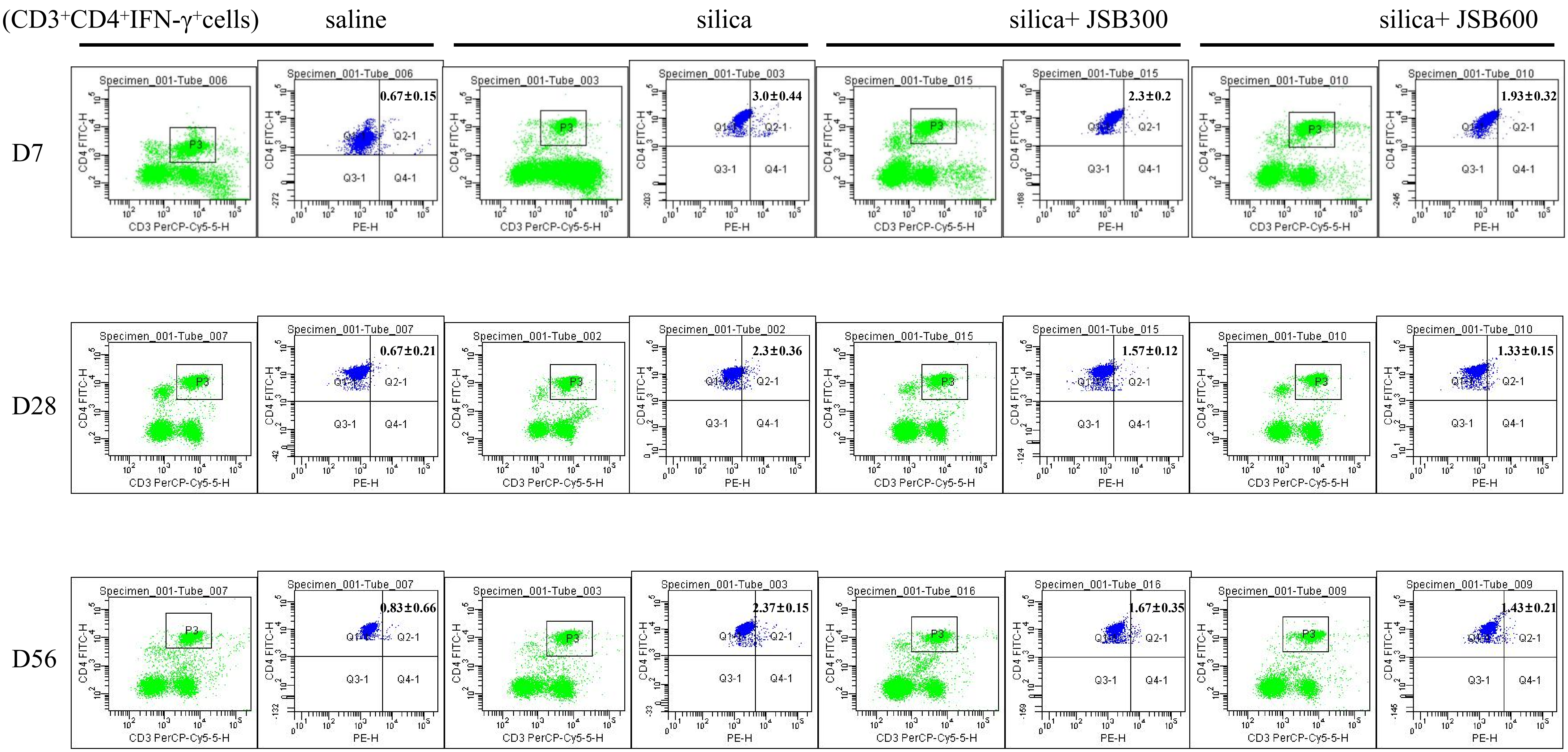

Figure S1: The detection of Th1 ($CD3^+CD4^+IFN\text{-}\gamma^+$) cells in peripheral blood lymphocytes by flow cytometry

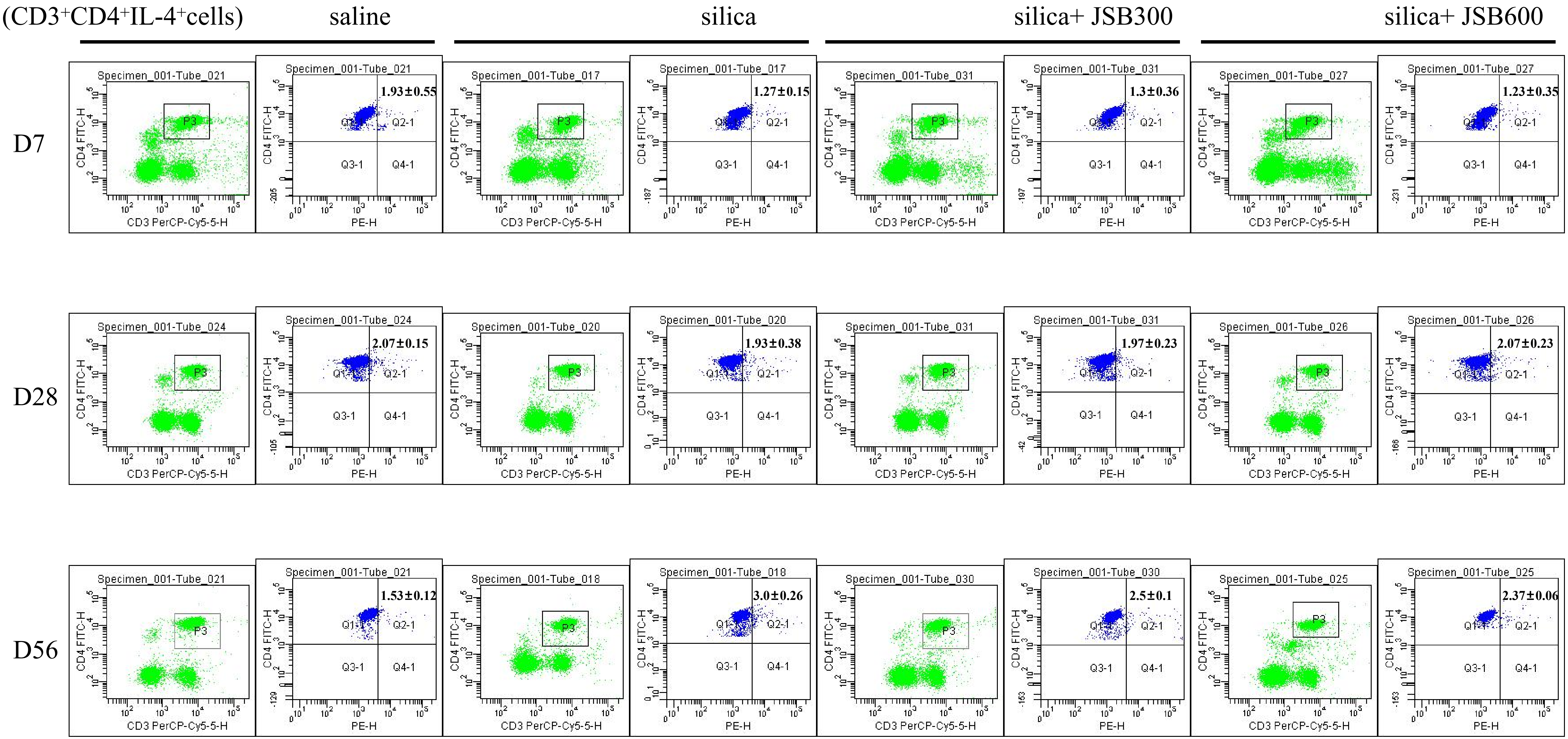

Figure S2: The detection of Th2 ($CD3^{+}CD4^{+}IL\text{-}4^{+}$) cells in peripheral blood lymphocytes by flow cytometry

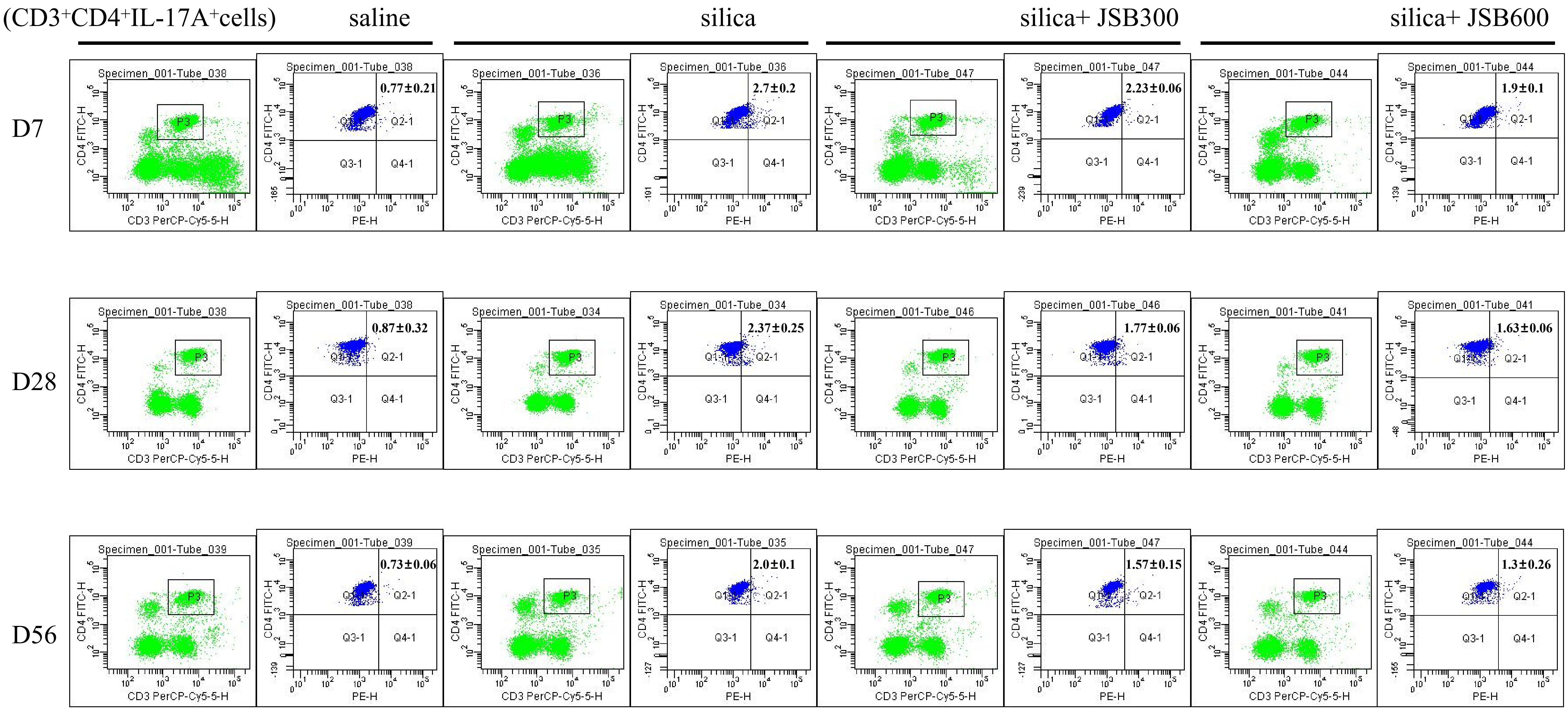

Figure S3: The detection of Th17 ($CD3^{+}CD4^{+}IL\text{-}17A^{+}$) cells in peripheral blood lymphocytes by flow cytometry

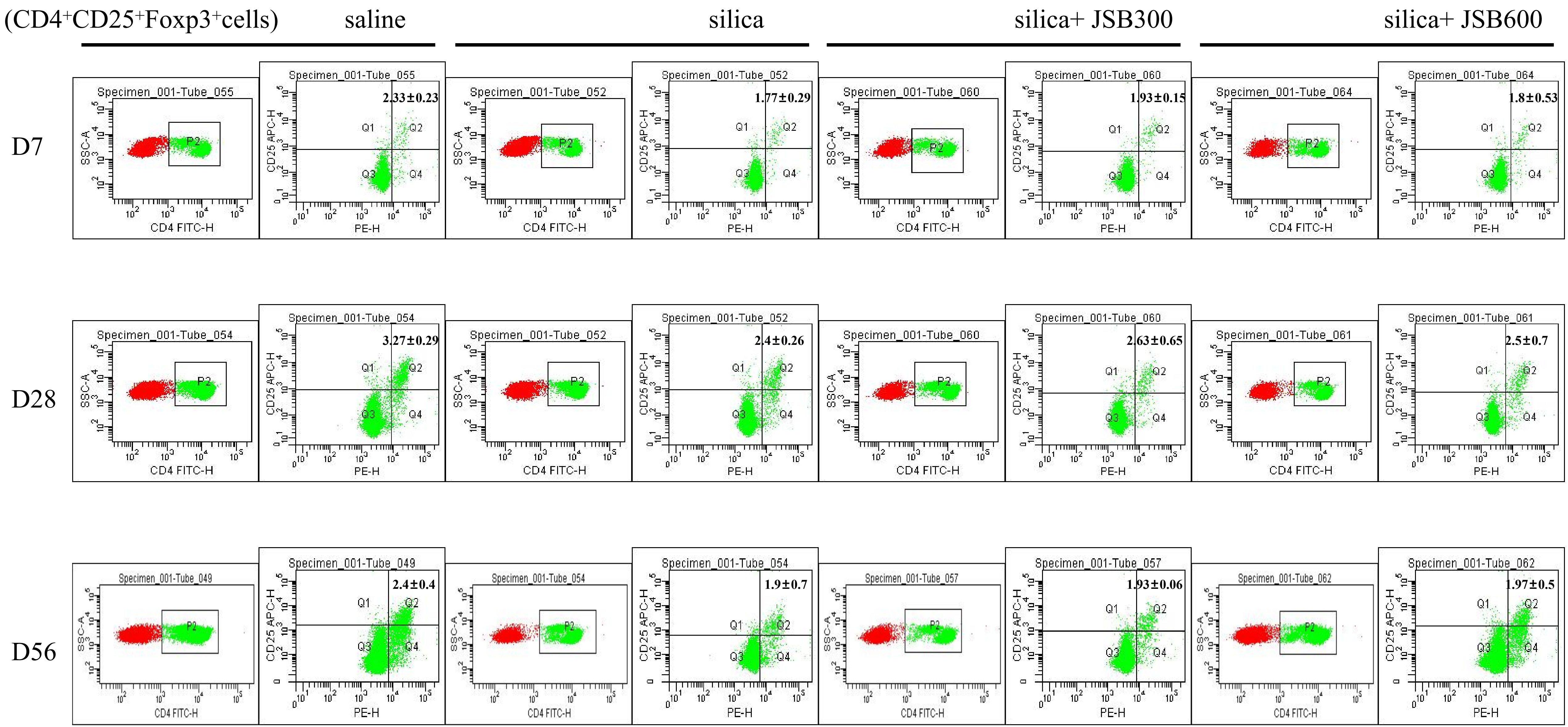

Figure S4: The detection of Treg ($CD4^+CD25^+Foxp3^+$) cells in peripheral blood lymphocytes by flow cytometry
